# Anti-HBV treatment partially restores the dysfunction of innate immune cells and unconventional T cells during chronic HBV infection

**DOI:** 10.3389/fimmu.2025.1611976

**Published:** 2025-07-04

**Authors:** Yiwen Shu, Sumeng Li, Yanqin Du, Xin Zheng

**Affiliations:** ^1^ Department of Infectious Diseases, Union Hospital, Tongji Medical College, Huazhong University of Science and Technology, Wuhan, China; ^2^ Institute of Infectious Diseases and Immunity, Union Hospital, Tongji Medical College, Huazhong University of Science and Technology, Wuhan, China

**Keywords:** hepatitis B virus, antiviral treatment, dendritic cell (DC), monocyte, natural killer (Nk) cell, MAIT (mucosal-associated invariant T) cell, γδT cell, NKT (natural killer T) cell

## Abstract

Despite the successful implementation of prophylactic vaccines, hepatitis B virus (HBV) continues to affect over 350 million individuals globally. It remains a predominant etiology of end-stage liver pathologies, including liver cirrhosis and hepatocellular carcinoma (HCC). While nucleos(t)ide analog (NUC) therapies effectively suppress viral replication, functional cure is achieved in less than 1% of patients annually. Given that viral clearance fundamentally requires reconstitution of antiviral immunity, emerging therapeutic paradigms necessitate combinatorial strategies integrating direct-acting antiviral agents with immunomodulatory interventions. Substantial research efforts have been directed toward elucidating the immunological mechanisms underlying HBV persistence during chronic infection. This review systematically summarizes the functional impairment of innate immune populations and unconventional T cell subsets across distinct clinical phases of chronic HBV infection, and characterizes longitudinal immune reconstitution patterns following antiviral treatments. Our review identifies potential immunological biomarkers and provides a mechanistic framework for developing targeted immunotherapies to achieve durable HBV control.

## 1 Introduction

Hepatitis B virus (HBV) remains a major global health challenge, chronically infecting an estimated 296 million people worldwide (1). Persistent HBV infection poses a significant risk for progression to end-stage liver diseases including cirrhosis, liver failure, and hepatocellular carcinoma. Current first-line antiviral therapies comprise two distinct modalities: pegylated interferon-α (PEG-IFN-α) and nucleos(t)ide analogs (NUCs). While PEG-IFN-α demonstrates the potential to induce HBsAg seroclearance in 10-30% of patients within defined treatment durations, its clinical utility is constrained by frequent severe adverse effects and the necessity for subcutaneous administration (2). In contrast, NUCs have gained widespread acceptance due to their oral dosing regimen and favorable safety profile. Despite these advantages, NUCs exhibit limited efficacy in achieving functional cure (defined as HBsAg loss) and require careful clinical management. Premature treatment discontinuation may trigger virological relapse with subsequent hepatic flares, and prolonged therapy raises concerns about indefinite or even lifelong medication dependency.

Emerging evidence suggests that sustained virological responses via antiviral treatments are accompanied by dynamic modulations of immune cell phenotypes and functional states (3). Notably, the interplay between antiviral therapy and immune reconstitution remains incompletely characterized, particularly regarding innate immunity components and unconventional T cell populations. This review systematically summarized current knowledge on the immunomodulatory effects of NUCs and PEG-IFN-α on temporal changes in innate immune cells (including NK cells, macrophages, and dendritic cells) and unconventional T cell responses during treatment. By integrating these findings, we aim to identify possible immune intervention for HBV immune therapy.

## 2 Partial functional recovery of innate immune cells following antiviral therapy

The innate immune system serves as the critical first line of defense against pathogens and plays a pivotal role in initiating and shaping subsequent adaptive immune responses. Beyond direct antiviral effector functions, innate immune cells are essential for antigen presentation, cytokine production and modulating the activation and function of HBV-specific T and B lymphocytes (4). While extensive researches have focused on the dysfunction and restoration of adaptive HBV-specific immunity during antiviral therapy (5), the longitudinal dynamics and functional reconstitution of innate immune cells remain relatively less explored. A deeper understanding of how current antivirals impact these innate compartments is crucial for revealing potential mechanisms to break immune tolerance and achieving functional cure.

### 2.1 Dendritic cells

Dendritic cells (DCs), as professional antigen-presenting cells, play a pivotal role as critical mediators bridging innate and adaptive immunity. Human DCs are broadly categorized into three main types, including monocyte-derived DCs (moDCs), plasmacytoid DCs (pDCs), and conventional DCs (cDCs) (6). MoDCs, characterized by the surface markers CD14, FcγRI (CD64), and FcεRI, become activated primarily under inflammatory conditions (7). In contrast, pDCs are identified by their expression of CD123, CD303 (BDCA2), and CD304 (BDCA4). These cells specialize in robust type I interferon (IFN-I) production in response to single-stranded viral RNA and DNA, a function mediated through pattern recognition receptors (PRRs) such as Toll-like receptor (TLR)-9 (8). The cDC population, often referred to as myeloid DCs (mDCs) in literature, consists of two principal subsets: cDC1s and cDC2s. cDC1s express CD141 (BDCA3) and excel at cross-presenting exogenous antigens on MHC class I molecules to activate CD8^+^ T cells. Conversely, cDC2s, which express CD1c/BDCA1 and CD172a, primarily present antigens on MHC class II molecules to stimulate CD4^+^ T cells (7).

Emerging evidence reveals profound DC dysfunction during chronic HBV infection, with distinct pathophysiological manifestations across disease phases. Studies demonstrate reduced mDC frequencies alongside elevated B7-H1 (PD-L1) expression on mDCs in chronic hepatitis B (CHB) patients (9, 10). Similarly, decreased peripheral pDC percentages, reduced TLR9 expression, and impaired CpG-induced IFN-α responses are observed in CHB patients compared to healthy controls (HCs) (11–15). Notably, Ouaguia et al. have reported higher pDC frequencies in CHB livers than those in HCs, while liver cDCs remain comparable (16). This dysfunction extends to disrupted crosstalk between pDCs and natural killer (NK) cell, evidenced by impaired cytotoxic activation of NK cells in CHB patients (17). Beyond classical DC subsets, recent studies by Li et al. have identified expanded circulating follicular DCs (FDCs; CD14^+^ CD21^high^)in chronic HBV patients compared to HCs (18).

Both circulating and intrahepatic cDC2s from HBV-infected patients exhibit reduced CD40/CD80 expression, whereas peripheral and hepatic pDCs display elevated CD40 levels compared to HCs (16). Altered expression of co-stimulatory/co-inhibitory molecules on DCs is prominent in CHB that co-stimulatory molecules (OX40L and 4-1BBL) are downregulated on peripheral pDCs and cDC1s, while PD-L1 expression on cDC2s and pDCs inversely correlates with HBV DNA (16). CD86 expression on pDCs is elevated in both immune-tolerant (IT) and immune-active (IA) phases compared to controls, with IA patients showing higher CD86 levels and enhanced IFN-α2 production (19). In addition, TGF-β1 significantly elevate within intrahepatic cDC2s and pDCs of IT patients compared to other disease stages or HCs (20). Metabolic disturbances are also evident, as Dumolard et al. have recently demonstrated dysregulated glycolysis and oxidative phosphorylation (OXPHOS) in hepatic cDC1s and pDCs across HBV infection stages (20). Furthermore, peripheral DCs from IT patients show significantly reduced levels of free cholesterol, lipid rafts, and LDL receptor (LDLR) compared to HCs. This lipid raft impairment, potentially influenced by HBsAg, can be partially restored by lipophilic statins, which also enhances the antigen-presentation ability of DCs. (21). Improtantly, functional recovery of DCs emerges in disease resolution phases, with inactive carriers (IC) demonstrating superior DC functionality over IT patients through increased expression of CD80, CD86, HLA-DR and IL-12 (22).

Functional impairments are further highlighted by TLR stimulation assays. Chronic HBV patients show significantly reduced production of IL-12p40/70 and TNFα by cDC2s, IFNα/TNFα/IFNλ1 by pDCs, and IFNλ1/TNFα/IL-12p40/70 by cDC1s compared to HCs (16). In contrast, intrahepatic DCs from CHB patients retain full functionality upon TLR triggering, producing pro-inflammatory cytokines at levels comparable to HCs (16). Furthermore, study on purified peripheral moDCs from CHB patients reveals heightened activation. The expression of both MHCII and co-stimulated molecules (CD80, CD86) as well as the cytokines (TNF-α, IL-10, IL-12) secretion in the purified peripheral moDCs from CHB patients are significantly higher than those from HCs when co-cultured with supernatant of HepG2.2.15 cells (23). Interestingly, enhanced autophagy is also observed in mo-DCs from chronic HBV patients compared to healthy donors upon re-exposure to HBV (23).

The immunomodulatory effects of antiviral therapies on DC populations exhibit substantial heterogeneity across clinical studies (**Table 1**). One study has illustrated that entecavir (ETV) therapy significantly reduces B7-H1 expression on peripheral DCs in CHB patients through suppression of HBcAg-mediated AKT/ERK/p38 signaling pathways (9). Furthermore, another study has shown that ETV induces early pDC proliferation (12-24 weeks), while CD86 is downregulated on pDCs in HBV DNA non-responders (27). Six-month therapy of adefovir dipivoxil (ADF) restores mDC frequency and enhances their capacity to produce TNF and IL-12, whereas the frequency and TNF-α and IL-10 secretion of pDCs remain refractory (26).

**Table 1:** Phenotypic and functional alterations of DCs in CHB patients during antiviral therapies.

Population study type	Intervention	Clinical outcome	Key immunological findings
n=63 HBeAg+ CHB Cohort (24)	PEG-IFN-α-2a (24 weeks)	Functional cure: 17/63	• Significant increase in pDC% and CD86 MFI vs baseline in both functional cure and non-cure groups • No intergroup difference in DC alterations
n=178 HBeAg+ CHB Cohort (25)	recombinant type I IFN-α(48weeks)	Responders (DNA undetectable, HBsAg↓): 77/178	• Responders showed elevated BDCA-2, ILT7 and TLR9 mRNA in pDCs vs non-responders • Positive correlation between DC activation markers and treatment response
n=12 CHB Cohort (26)	ADF (6 months)	Rapid decrease in HBV DNA and normalization of ALT within 3 months	• Persistent reduction of pDC% without post-treatment recovery • Dichotomous cytokine response of pDC: ↓TNF-α vs ↑IFN-α, IL-10 at 6 months • Enhanced mDC function: ↑TNF, IL-12 production
n=87 HBeAg+ CHB (PEF-IFN-α-2a:48; ETV:39) Cohort (27)	PEG-IFN-α-2a or ETV (48 weeks)	PEG-IFN responders (33/48): HBsAg decreased > 60% in 48 weeks ETV responders (25/39): undetectable HBV DNA in 48 weeks	• PEG-IFN responders: ↑CD86^+^ pDC% correlated with HBsAg decline • ETV non-responders: ↓CD86^+^ pDC% associates with persistent HBV DNA
n=16 HBeAg+ CHB Cohort (9)	ETV (6 months)	ALT/AST and HBV-DNA levels decreased	• Pre-treatment: ↓DCs%, mDCs% and ↑B7-H1 vs healthy controls • Post-treatment: ↓B7-H1 expression on DCs
n=14 HBV-IA Cohort (28)	LAM (6 months)	HBV DNA undetectable, ALT normalization	• HBeAg seroclearance associates with: - ↑Circulating pDCs at 180 days - Restored PBMC IFN-α production capacity
n=48 CHB (24 HBeAg+) on NUCs Phase II RCT (29)	Oral selgantolimod (TLR8 agonist) 3 mg, 1.5 mg, or placebo once weekly (24 weeks)	• Only selgantolimod-treated patients (n=39) had HBsAg declines greater than 0.1log_10_ IU/ml at weeks 24 (7/39) and 48 (10/39). • HBsAg loss (2/39 through 48 weeks), HBeAg loss (3/19 through 48 weeks).	• ↑in the selgantolimod group with a dose-dependent trend

CHB, chronic hepatitis B; HBV, hepatitis B virus; PEG-IFN-α, pegylated interferon alpha; MFI, mean fluroscence indensity; pDC, plasmacytoid dendritic cell; mDC, myeloid dendritic cell; PBMC, peripheral blood monomuclear cell; ADF, adefovir; ETV, entecavir; LAM, lamivudine; IA, immune active; ALT, alanine aminotransferase; AST, aspartate transaminase; NUC, nucleos(t)ide analog; RCT, random controlled trial.

↑, increase; ↓, decrease.

Interferon-based regimens reveal distinct immunostimulatory patterns. PEG-IFN-α-2a treatment induces sustained CD86 upregulation on pDCs in patients achieving functional cure (24). Moreover, the frequency of pDC increases at week 24 post-treatment in the functional cure group (24). Consistently, Cao et al. have found that HBsAg decline significantly associates with CD86 elevation on pDCs during IFN-α treatment (27). Mechanistically, IFN-α treatment enhances hepatic pDC expansion and upregulates TLR-9 mRNA in peripheral blood mononuclear cells (PBMCs) of virological responders (25). A recent clinical trial of selgantolimod (TLR8 agonist) has demonstrated significant increase of peripheral pDCs, with a dose-dependent trend (29). Taken together, critical analysis identifies three determinants of DC functional restoration, including baseline DC subset characteristic, different antiviral agents, and variant duration of treatment and observation.

### 2.2 Monocytes

Monocytes, originating from common myeloid progenitors (CMPs) in the bone marrow, constitute approximately 10% of human peripheral leukocytes and perform multifaceted functions in homeostasis and inflammation (30). In humans, two functionally distinct subsets are recognized. CD14^++^ CD16^-^ “migratory” monocytes are capable of tissue infiltration, and CD14^+^ CD16^+^ “patrolling” monocytes maintain vascular surveillance (31).

Chronic HBV exposure induces immunoregulatory reprogramming of monocytes. Monocytes from chronically infected individuals demonstrate elevated expression of TNF-α, IL-10, TGF-β, PD-L1, Gal-9 and HLA-E compared to HCs (32, 33). Notably, PD-L1 upregulation on monocytes is particularly pronounced in HBeAg-positive patients (34, 35). Furthermore, the hepatic compartment of chronic HBV (CHB) patients shows an enrichment of monocytes expressing Gal-9 and PD-L1 compared to HCs (33). Functionally, monocytes from IT patients and HBeAg-positive or -negative CHB patients demonstrate suppressed signaling through TLR2, TLR4, and TLR9 compared to ICs and HCs. This functional impairment is accompanied by reduced production of IL-12, TNF-α, and IL-6, as well as diminished phagocytic capacity and oxidative response (36–38). Moreover, PD-L1- and Gal-9-expressing monocytes in CHB contribute to the dysregulation of both adaptive and innate immune responses (33). Another study has revealed significantly downregulated expression of membrane-bound CD163, a monocyte activation marker, on circulating monocytes from both treatment-naïve CHB patients and those achieving HBsAg loss compared to HCs (37). Conversely, circulating soluble CD163 (sCD163) levels are elevated in CHB patients with significant inflammation (A≥2) or fibrosis (F≥2) (37).

Emerging evidence suggests antiviral interventions may partially reverse HBV-induced monocyte dysfunction, though therapeutic outcomes remain heterogeneous. After one year of treatment, tenofovir disoproxil fumarate (TDF) fails to restore monocyte functionality, as evidenced by unchanged monocyte subset distribution and proportions expressing PD-LI, Gal-9, TLR-2, IL-12, IL-10, CD64, and iNOS before and after treatment (33, 38), whereas responders to Peg-IFN-α and ETV demonstrate partial TLR9 expression recovery on monocytes (36). Intrahepatic transcriptomics reveal elevation of hepatic monocytes after 24-week PEG-IFN-α treatment (39). Recent single-cell analyses reveal that PEG-IFN-α reduces proportions of pro-inflammotory CD14^+^ and CD16^+^ monocytes, accompanied by systemic immune reprogramming from TNF-α-dominant to IFN-α-driven transcriptional profiles (40). Consistently, NUC-treated patients exhibit upregulated expression of TLR-associated genes LY6E and STK4 on monocytes compared to ICs (41). A recent clinical trial of selgantolimod (TLR8 agonist) has demonstrated significant increase of peripheral CD14^+^ classical monocytes, with a dose-independent trend (29). Collectively, these findings position monocytes as pivotal mediators of HBV immunopathogenesis. While current antivirals show partial efficacy in reversing monocyte dysfunction, stratified interventions targeting subset-specific reprogramming are needed to achieve functional cure.

### 2.3 Myeloid-derived suppressor cells

Myeloid-derived suppressor cells (MDSCs), constituting less than 1% of myeloid cells in healthy individuals (42), are a heterogeneous population of immunosuppressive myeloid cells comprising two functionally distinct subsets, polymorphonuclear MDSCs (PMN-MDSCs) and monocytic MDSCs (M-MDSCs) (43, 44). In human PBMCs, these subsets are phenotypically characterized as CD11b^+^ CD14^−^ CD15^+^/CD66b^+^ (PMN-MDSC) and CD11b^+^CD14^+^HLA-DR^−/lo^CD33^+^CD15^−^ (M-MDSC) (45). MDSCs undergo significant expansion under pathological conditions, suppressing T cell responses and promoting disease progression through multiple mechanisms (46).

Several clinical studies demonstrate remarkable expansion of circulating MDSCs in CHB patients compared to HCs (47–49) The frequency of MDSCs positively correlates with HBV DNA load, HBeAg levels and HBsAg levels (48, 50). Both M-MDSC and granulocytic-MDSC (gMDSCs) from different phases of CHB expressed high TGF-β and IL-10 (51). Notably, purified M-MDSCs from HBeAg-positive patients exhibit enhanced suppression of CD4^+^/CD8^+^ T cell proliferation and IFN-γ production compared to those from HBeAg-negative individuals (52). Moreover, gMDSCs expressing arginase expand during high viral replication phases, impairing T cell function via arginase-dependent pathways (53). Notably, an enrichment of PD-L1/Arg/iNOS expressing hepatic MDSCs is observed in CHB patients compared to HCs (51). A recent single-cell RNA sequencing of PBMC has shown that a CD14^+^ cluster with an MDSC-like phenotype predominantly accumulates in patients with CHB, with high expression of genes with immunoregulatory functions (54).

Apart from peripheral immune suppression, MDSCs also contribute to central tolerance via chemokine-mediated trafficking. HBsAg upregulates CCR9 expression on M-MDSCs through ERK1/2-IL-6 signaling, facilitating thymic homing via CCL25 chemotaxis (55). This process enables peripheral HBsAg transport to thymic medulla, ultimately inducing clonal deletion of HBsAg-specific CD8^+^ thymocytes, a mechanism predominant in pediatric CHB patients (55). Collectively, these findings unveal MDSCs as central orchestrators of HBV-induced immune tolerance through peripheral and thymic mechanisms, offering potential targets for therapeutic intervention.

Current evidence suggests suboptimal efficacy of NUCs in reconstituting MDSC homeostasis. One-year TDF monotherapy fails to restore MDSC frequency and the secretion of IL-10 and TGF-β or improve HBV-specific T-cell responses (51, 56). Strikingly, patients achieving functional cure through PEG-IFN-α-2a display substantial M-MDSC reduction (57). Consistently, targeting MDSCs with all-trans retinoic acid restores HBV-specific CD4^+^ and CD8^+^ T cell proliferation and IFN-γ production in CHB patients (50).

### 2.4 NK cells

As critical effectors of innate immunity, NK cells mediate rapid antiviral and antitumor responses. In humans, NK cell populations are traditionally classified into CD56^dim^ (cytotoxic) and CD56^bright^ (immunoregulatory) subsets based on CD56 and CD16 surface marker expression (58). NK cells exhibit dual roles in HBV immunity, balancing antiviral defense mechanisms and immunopathogenic potential through liver injury (59). During acute HBV infection (AHB), peripheral CD56^bright^ NK cells undergo significant expansion (60) and display an activated phenotype characterized by upregulated activation receptors (NKp30, NKp44, NKp46 and NKG2C), activation markers (CD38 and HLA-DR), and cytotoxic mediators like TRAIL, alongside downregulation of inhibitory receptors (CD158a/b and NKG2A) (61). Elevated CD107a expression and robust IFN-γ production upon IL-12+ IL-18 or K562 stimulation have also been observed in peripheral CD56^bright^ NK cells during acute HBV (61). Notably, CD56^dim^ NK cell-mediated antibody-dependent cellular cytotoxicity (ADCC) associates with early HBsAg clearance in AHB (62). Temporal analyses, however, reveal transient suppression of IFN-γ and TNF-α production during peak viremia, with functional recovery upon viral resolution (63).

In chronic HBV infection, phenotypic and functional defects of NK cells are well-documented. Discrepancies in circulating NK cell frequencies across studies reflect population heterogeneity and clinical phase variations (64–66). Progressive NK cell dysfunction has been observed in chronic HBV infection, characterized by reduced expression of activating receptors (e.g. NKG2D), increased inhibitory checkpoint molecules (PD-1, Tim-3, CD94) (67), with the frequency of intrahepatic PD-1^+^ NK cells being the highest in HBeAg+ HBV patients (68). This dysfunction is further marked by attenuated antiviral cytokine (IFN-γ, TNF-α) secretion (69, 70), and elevated immunosuppressive IL-10/TGF-β1 production (71, 72). Conversely, NK cells may negatively regulate HBV-specific T cells through TRAIL-R2-mediated lysis (73). Furthermore, the activation of NK cells driven by proinflammatory cytokines (IFN-α, IL-12, IL-15, IL-8) also exacerbates liver inflammation via NKG2D/TRAIL/IFN-γ-mediated hepatocyte damage, particularly in IA phase (64, 74–76). This pathogenic role is supported by a positive correlation between intrahepatic NK cell accumulation and histological inflammation severity (77). Furthermore, TRAIL expression on CD56^bright^ NK cells positively correlates with liver inflammation and ALT flare (65, 71, 75). Intrahepatic analyses of a recent single-cell RNA sequencing demonstrate that the CXCR6+ NCAM1+ CD160^high^ liver-resident NK-cell cluster with a significant higher expression of IL-32 within the HBsAg-high group compared to HBsAg-low group (78).

Functional analyses reveal discrepancies in NK cell cytotoxic activity. While NK cells from IA patients exhibit enhanced TNF-α, IFN-γ, and CD107a production compared to HCs (75, 79), cytokine-mediated functional exhaustion has been reported following IL-2 and IL-12 or IL-21 stimulation (71, 80, 81). Conversely, other studies illustrate preserved cytotoxic function of NK cells, as evidenced by intact K562 lysis capacity (82, 83).

Emerging evidence reveals the multifaceted immunomodulatory effects of antiviral therapies on NK cells in CHB (**Table 2**). NUCs and PEG-IFN-α therapies have demonstrated marked heterogeneity across studies regarding capacity to reshape NK cell quantity, phenotype, and function, influenced by treatment duration, therapeutic agents, and patient-specific factors. A recent randomized controlled trial has observed significant upregulation of activation markers (TRAIL, HLA-DR, Ki-67, CD38) and receptors (NKp46, NKG2D, NKp30, NKG2A) on total NK cells—irrespective of HBsAg decline magnitude (98). However, some studies report transient expansion of immunoregulatory CD56^bright^ subset during NUC therapy, with normalization post-HBsAg clearance (88, 96), while other investigations document static or even reduced NK cell counts in NUC-treated cohorts, including telbivudine (LDT) and ETV (81, 97). Intrahepatic transcriptomics reveal no alteration of hepatic NK cells after 24-week PEG-IFN-α treatment (39).

**Table 2:** Functional and phenotypic alterations of NK cells during anti-HBV therapies.

Population study type	Intervention	Clinical outcome	Key immunological findings
64 treatment-naïve vs 22 treated CHB Case-control (71)	LAM+ADF combination therapy	Treated group: HBV DNA undetectable	• ↓CD56^bright^ subset proportion to HC levels • ↓TRAIL expression (normalization) • Partial recovery of IFN-γ production in CD56^dim^ subset (remained ↓vs HC)
5 treated, 42 active, 21 inactive CHB Case-control (84)	IFN-α1b + ADF	HBV DNA reduction	• ↓NKG2A^+^ NK% with HBV DNA reduction • NKG2A expression inversely correlated with viral load
n=15 active CHB Cohort (81)	ETV (6 months)	HBV DNA reduction	• Preserved total NK count • CD56^bright^: ↑CD69 expression (2-fold) • Both subsets: ↓NKG2A • ↑IFN-γ^+^ NK cell frequency
n=18 HBeAg+ CHB Cohort (85)	ETV (24 weeks)	HBV DNA/HBsAg/HBeAg reduction; ALT/AST decrease	• Stable NK cell numbers • ↓Activation markers: NKG2D, NKp30, CD107a
n=30 HBeAg+ suboptimal responders to ADV Cohort (86)	Switch to ETV (6 months)	HBV DNA/HBsAg reduction; HBeAg seroconversion (11/30); ALT/AST decrease	• ↑Total NK cell count (normalization) • ↑CD244^+^ activated NK cells to HC levels
n=54 active CHB Cohort (87)	LDT (13 months)	HBV DNA reduction; HBeAg seroconversion (15/54); ALT/AST normalization	• Gradually ↑NK cell count • ↑CD244^+^ activated NK% (time-dependent, reaching HC levels)
n=52 IA patients Cohort (88)	LDT (48 weeks)	HBV DNA/HBsAg reduction; HBeAg seroconversion (11/52); ALT decrease	• ↑CD56^bright^ NK% • ↑Activating receptors: NKG2D, NKp46 on CD56^bright^ • ↓Inhibitory receptor NKG2A on CD56^bright^
n=14 CHB Cohort (65)	PEG-IFN-α-2a +ADF (48 weeks)	Responder: HBsAg loss at week 72 (7/14)	• ↑NK cell proportion • ↑CD56^bright^/CD56^dim^ ratio • ↑Activation markers: Ki67, HLA-DR, CD38, NKp30, NKp46 on both subsets • Baseline predictors: ↓CX3CR1 (CD56^bright^), ↓NKG2A (CD56^dim^) • ↑TRAIL^+^ and IFN-γ^+^ NK in responders
n=55 HBeAg+ CHB (27 IFN-switch vs 28 on-ETV) RCT (89)	ETV→PEG-IFN-α vs continued ETV (48 weeks)	• IFN-switch group: HBeAg loss (21/27) HBsAg loss (4/27) • on-ETV group: HBeAg loss (16/28) HBsAg loss (0/28)	• IFN-switch group vs on-ETV group: - ↑CD56^bright^ % - ↑NKp30^+^/NKp46^+^ CD56^bright^ - ↑TRAIL, TNF-α, IFN-γ production on CD56^bright^
15 treated vs 69 active CHB Case-control (90)	ETV (6 months)	–	• ↓NKG2A on NK post-treatment
n=20 pediatric HBeAg+ CHB Cohort	PEG-IFN-α (48 weeks)	Complete responder: HBsAg seroconversion at week 48-96 (11/20)	• Complete responders: ↑TRAIL on CD56^bright^
n=24 CHB (12 TDF vs 12 ADV) RCT (91)	TDF/ADV (24 weeks)	HBV DNA reduction	• Both groups: ↓NKG2A, ↓KIR2DL3 on NK • TDF group: ↑NK cells • ADV group: ↑CD158b^+^ NK
87 pregnant IT (41 untreat vs 46 TDF) Case-control (92)	TDF (32-week gestation to delivery)	–	• Antepartum: ↑Total NK% and NKp46^+^ NK vs untreated
n=101 CHB (51 naïve; 50 IFN-plateau (HBsAg reduction<0.5 lg IU/mL) Cohort (93)	PEG-IFN-α (initial vs interrupted-resumed) (24 weeks)	–	• Initial group: ↓CD56^dim^ %; ↓ (CD57, TIGIT) on CD56^dim^ NK • Plateau group: ↑CD57 on CD56^dim^ NK after IFN interruption
n=66 HBeAg+ CHB Cohort (94)	PEG-IFN-α-2a (24-48 weeks)	Functional cure (17/66)	• Functional cure group: - ↑CD56^bright^ % - ↑NKp46^high^ % and MFI on NK - ↑IFNAR2 MFI on NK • Non-cure: Only NKp46 MFI ↑ on NK
n=89 HBeAg+ CHB (49 IFN, 40 ETV) Cohort (95)	PEG-IFN-α/ETV (48weeks)	• PegIFN group: responder (HBsAg reduction>60%, 33/49); HBV DNA undetectable (45/49); HBeAg seroconversion (9/49) • ETV group: HBV DNA undetectable (27/40); HBeAg seroconversion (3/40)	• PegIFN group: - ↑Total NK, CD56^bright^, NKp46^+/bright^ NK (↑↑ in responders) - HBsAg decline correlates with NKp46^bright^ NK at baseline/wk12 • ETV group: ↑NK at wk12/24
n=71 HBeAg- CHB 25 naïve vs 46 NUC-treated (10/46 HBsAg clearance) Case-control (96)	NUCs	–	• ↑NK cells after NUC (significant post-HBsAg clearance) • HBsAg clearance: ↓CD56^bright^ to HC levels • ↓TRAIL/CD38/Ki67 after viral suppression and ALT normalization
n=41 CHB (ALT 2-5×ULN) Cohort (97)	LDT (36 weeks)	HBV DNA reduction; ALT/AST decrease	• No significant NK frequency changes
n=53 HBeAg- CHB on NUC RCT (98)	25 PEG-IFN-α v.s 28 NUC (48weeks)	HBsAg Log10 decline> 0.5 (n=12); HBsAg Log10 decline< 0.5 (n=13)	• ↑TRAIL, HLA-DR, Ki-67, CD38 on total NK in both groups • ↑NKp46, NKG2D, NKp30, NKG2A on total NK in both groups
n=28 HBeAg- CHB on NUCs (3-4 yrs) RCT (99)	GS-9620 (TLR7agonist) (12 weeks) at 1/2/4 mg/w doses	HBsAg show no significant reduction in patients given any dose of GS-9620.	• ↑ total and CD56^bright^ NK cells • ↑CD69, HLA-DR, TRAIL on CD56^bright^ and CD56^dim^ NK cells across all doses • ↑ IFN-γ, TNF-α and CD107a of NK • ↓NK cell-mediated inhibition of HBV-specific T cells
n=14 CHB Phase 1b RCT (100)	a single 3mg dose of selgantolimod (TLR8 agonist)	–	• ↑CD69 on NK cells 8 hours post-administration
n=27 CHB Phase I/II RCT (101)	α-GalCer at doses of 0.1/1/10 ug/kg All received 3 doses (week0, 4, 8)	No clearly affect HBV DNA and ALT levels	• ↓NK cells at 0.1 and 1 μg/kg doses ↑NK cells at 10 μg/kg dose • ↑CD69 on NK in all treatment groups
n=48 CHB (24 HBeAg+) on NUCs Phase II RCT (29)	Oral selgantolimod (TLR8 agonist) 3 mg, 1.5 mg, or placebo once weekly (24 weeks)	• Only selgantolimod-treated patients (n=39) had HBsAg declines greater than 0.1log_10_ IU/ml at weeks 24 (7/39) and 48 (10/39). • HBsAg loss (2/39 through 48 weeks), HBeAg loss (3/19 through 48 weeks).	• No change of NK frequency in the selgantolimod group

ADF, adefovir; ADV, adefovir dipivoxil; ALT, alanine aminotransferase; AST, aspartate aminotransferase; CHB, chronic hepatitis B; ETV, entecavir; HC, healthy controls; IA, immune-active; IT, immune-tolerant; LAM, lamivudine; LDT, telbivudine; MFI, mean fluorescence intensity; NUC, nucleos(t)ide analog; PEG-IFN, pegylated interferon; RCT, random controlled trial; TDF, tenofovir disoproxil fumarate; TRAIL, TNF-related apoptosis-inducing ligand; ULN, upper limit of normal.

↑, increase; ↓, decrease.

The phenotype of NK cells varies among different studies following antiviral therapy. Inhibitory receptors such as NKG2A and KIR2DL3 demonstrate progressive downregulation in tandem with viral suppression under NUC therapy (88, 90). Consistently, activation receptors(NKp30, NKp46, and NKG2D) exhibit temporal upregulation patterns that parallel HBsAg clearance trajectories (88, 92, 94, 95). ETV monotherapy transiently suppresses NKG2D and NKp30 expression on NK cells in HBeAg-positive patients (85), whereas therapeutic regimen switching (ADV to ETV) enhances CD244^+^ activated NK subsets (86). PEG-IFN-α induces TRAIL upregulation on CD56^bright^ NK cells in complete responders (102), while LAM-ADV combination therapy restores TRAIL expression without rescuing IFN-γ production deficits in CD56^dim^ subsets (71). Intriguingly, ETV treatment enhances CD69 expression and IFN-γ production specifically within CD56^bright^ NK populations (81). Furthermore, PEG-IFN-α discontinuation in plateau-phase patients reduces exhaustion markers (CD57, TIGIT) on CD56^dim^ NK (93). Several clinical trials of novel immunotherapies exhibit prominent alteration on NK cells. GS-9620 (TLR-7 agonist) rapidly upregulates NK activation markers (CD69, TRAIL, HLA-DR) and enhances effector functions (IFN-γ, TNF-α, degranulation) (99). Preliminary research of selgantolimod (TLR-8 agonist) activates NK cells as well, evidenced by CD69 expression (100), while another phase II trial demonstrates no alteration in circulating NK cell frequencies (29). α-GalCer modulates NK cell frequencies bidirectionally (decreasing at lower doses, increasing at 10 μg/kg), and effectively increases CD69 expression (101). Collectively, these findings highlight the critical role of NK cells in antiviral immunity, with treatment-induced phenotypic remodeling potentially serving as a biomarker for therapeutic efficacy.

## 3 Effects of antiviral therapies on unconventional T cells

Unconventional T cells (UTCs) represent a heterogeneous group of non-classical MHC-restricted lymphocytes that recognize non-peptide, non-polymorphic antigens. This family includes γδ T cells, invariant natural killer T (iNKT) cells, mucosal-associated invariant T (MAIT) cells, and CD4/CD8 double-negative T cells (103). UTCs orchestrate rapid antimicrobial responses through producing potent cytokines (e.g. IFN-γ, TNF-α, IL-17) and exerting cytotoxicity during early infection phases, prior to conventional αβ T cell activation (104). Beyond pathogen defense, UTCs contribute to chronic inflammation and tissue homeostasis (105). UTCs account for 10–30% of peripheral T cell populations in adult (106). These cells predominantly reside at mucosal sites and notably enriched in the human liver, positioning them as key sentinels and early responders in HBV infection (107, 108). Despite their potential significance in hepatic immunity, the impact of chronic HBV infection and subsequent antiviral therapy on the frequency, phenotype, and function of distinct UTC subsets is less comprehensively characterized compared to conventional HBV-specific CD4^+^ and CD8^+^ T cells. Investigating the dynamics and restoration of UTCs during treatment is vital, as these cells may contribute uniquely to viral control, immunopathology, and offer novel immunological insights or biomarkers for therapeutic efficacy and the development of combined immunotherapies aimed at functional cure.

### 3.1 MAIT cells

MAIT cells are characterized by their semi-invariant TCR α-chain (usually Vα7.2–Jα33/12/20 in humans) and restriction to the MHC-I-related protein MR1 (103, 109), which presents microbial riboflavin (vitamin B2) and folate (vitamin B9) derivatives (110). MAIT cells constitute approximately 5% of circulating T cells (111) but are enriched in mucosal tissues, representing up to 45% of hepatic T lymphocytes (109). Upon activation, they predominantly secrete IFN-γ and TNF, with a minor subset producing IL-17A (109).

The frequency, phenotype and cytokine production of MAIT cells exhibits conflicting patterns across studies in CHB patients. Several studies have reported reduced circulating MAIT cells in CHB compared to HCs (112, 113), whereas another study documents comparable levels (114). MAIT cell reduction is also observed in patients with HBV-related acute-on-chronic liver failure (115). Mechanistically, this reduction potentially attributes to conjugated bilirubin-mediated apoptosis of MAIT cells (113). Several studies have documented the upregulation of activation markers (CD69, HLA-DR, CD38), immunosenescence marker CD57, and inhibitory receptors (PD-1, CTLA-4) on peripheral MAIT cells in CHB compared to HCs (113, 116, 117). However, another study demonstrates reduced expression of CD69 on MAIT cells in CHB patients (118). Notably, CD69 expression on MAIT cells correlates positively with HBV viral load, while inhibitory markers (PD-1 and CTLA-4) on MAIT cells show negative correlation with HBV DNA levels (115, 116). Further functional assessments show enhanced IFN-γ and Granzyme B secretion from MAIT cells in CHB patients than those in HCs upon anti-CD28/E.coli co-stimulation (114, 118), whereas combined stimulation of IL-12 and IL-18 yields impaired IFN-γ responses in CHB patients (119). Single-cell transcriptomics identify two hepatic MAIT subsets in CHB, T7(CD3^+^SLC4A10^+^TNFAIP3^+^) cells displaying proinflammatory cytokine secretion and immune cell recruitment capacities, and T6(CD3^+^SLC4A10^+^TNFAIP3^-^) cells with impaired antiviral function (120). The progressive shift toward T6 predominance during advanced hepatic inflammation highlights MAIT cell dysfunction in chronic HBV pathogenesis (120). These findings collectively illustrate the complex duality of MAIT cell responses in CHB, balancing protective immunity with inflammation-driven exhaustion. Longitudinal analyses suggest preserved MAIT cell frequencies during NUC therapy (114, 121). Nevertheless, treatment-induced normalization of CD38 activation marker expression implies partial recovery of MAIT cell functionality, though complete phenotypic and functional restoration remains to be established (114). A phase 1b clinical trial of selgantolimod (TLR8 agonist) shows the elevation of CD69 on MAIT after a single dose (100).

### 3.2 γδ T cells

γδ T cells are defined by their unique TCR consisting of a γ-chain and a δ-chain, which enables antigen recognition independent of MHC class I/II molecules (103). Two major subsets exist in humans, Vδ1^+^ and Vδ2^+^ γδ T cells (122). Vδ1+ cells, characterized by pairing of the Vδ1 chain with diverse Vγ family members (Vγ2/3/4/5/8/9) (123) predominantly reside in mucosal and epithelial tissues such as intestinal epithelium (124), skin (125, 126), spleen and liver. In contrast, Vδ2^+^ cells typically express an invariant Vγ9 chain paired with Vδ2 (127), constituting 50–95% of circulating γδ T cells in human peripheral blood (128, 129). These cells are activated through phosphoantigen recognition via butyrophilin 3A1 (BTN3A1) (130, 131), triggering rapid secretion of cytotoxic molecules and Th1 cytokines (IFN-γ and TNF-α) to combat malignancies and microbial pathogens (132, 133). Additionally, Vδ3^+^ T cells have been found in the periphery which only consist about 0.2% of γδ T cells, while in the liver they are more abundant. Limited studies on this subset show their capacity to secret Th1, Th2 and Th17 cytokines (134).

Acute HBV infection significantly reduces peripheral γδ T cell proportions and absolute counts compared to CHB and HCs, negatively correlating with serum ALT (135). AHB patients exhibit heightened activation profiles in circulating γδ T cells compared to HCs, characterized by upregulated CD38, HLA-DR, granzyme B, CD107a, and distinct transcriptional polarization as Tbet^+/hi^ Eomes^dim^ Vδ1 subsets and Tbet^dim^ Eomes^hi^ Vδ2 subsets (135, 136). Concurrently, intrahepatic γδ T cells accumulate in inflamed liver lobules during AHB (135), a phenomenon recapitulated in acute HBV murine models where hepatic γδ T cell expansion coincides with early-stage IFN-β production (137).

In chronic HBV infection, peripheral γδ T cells are significantly reduced in CHB patients relative to HCs (138), particularly in severe liver inflammation (ALT>3×ULN) (139). However, one study reports comparable γδ T cell frequencies between symptomatic CHB and HCs (140), and some studies document elevated Vδ1 T cell percentages in CHB (138, 141). Hepatic γδ T cells, particularly the Vδ2 subset, decrease in CHB patients, especially within the IA group (138). Analysis of paired samples further reveals markedly lower hepatic Vδ2 T cell levels than their peripheral counterparts in IA patients (138).

The phenotype and function of γδ T cells varies among different studies. Elevated exhaustion markers (PD-1, Tim-3 and Lag-3) and activation markers (CD69, CD38 and HLA-DR) levels are frequently reported in CHB (140, 142). Paradoxically, Chang et al. have observed decreased PD-1, CD38, Ki-67, Tim-3, and CD158a expression on Vδ2 T cells from CHB patients compared to HCs (136). Intriguingly, PD-1 expression on circulating Vδ2^+^ cells inversely correlates with serum 25(OH)D3 levels in CHB (142). PMA/ionomycin stimulation enhances IFN-γ/granzyme B/TNF-α co-expression on γδ T cells from CHB patients (136, 141). However, another study describes suppressed IFN-γ secretion of γδ T cells, but can be reversible by Tim-3/Lag-3 blockade (136, 140). Functional cytotoxicity assays reveal impaired γδ T cell-mediated lysis of HBV-infected hepatocytes in symptomatic CHB compared to HCs, though asymptomatic carriers retain partial cytolytic activity than symptomatic patients (139).

The impact of antiviral therapies on γδ T cell populations in CHB treatment presents complex immunological modifications (**Table 3**). A randomized controlled trial has revealed TDF/PEG-IFN-α combination therapy in HBV-suppression patients exhibits no significant alterations in γδ T cell frequencies or their functional capacity to produce IFN-γ/TNF-α/granzyme B/CD107a (143). Conversely, PEG-IFN-α monotherapy reduces γδ T cell numbers, accompanied by enhanced TNF-α/CD107a expression (144, 145). Furthermore, treatment responders exhibit distinct γδ T cell differentiation patterns characterized by transient early effector cell expansion and reduced T_em_ subsets (145). Longitudinal monitoring of LDT therapy suggests that elevated baseline CD4⁻CD8⁻ γδ T cells predict non-response and virologic relapse (146). Collectively, these findings underscore the heterogeneity of γδ T cell responses during CHB therapy, with dynamic changes in subsets and functional markers correlating with treatment efficacy and relapse risk.

**Table 3:** Alterations in γδ T cell profiles during anti-HBV therapies.

Population study type	Intervention	Clinical outcome	Key immunological findings
n=30 CHB (on TDF, virally suppressed): 10 add-on Peg-IFN-α; 20 on-TDF RCT (143)	TDF ± PEG-IFN-α (48 weeks)	Add-on group: HBsAg reduction	• add-on vs monotherapy -γδ T cells→ -IFN-γ^+^/TNF-α^+^/GrzB^+^/CD107a^+^ γδ T cells→
n=10 treatment-naïve CHB Cohort (144)	PEG-IFN-α (48 weeks)	Responder (5/10): ALT normalization + HBeAg loss+ HBV DNA reduction>3log_10_	• ↓ γδ T • ↑ TNF-α^+^/CD107a^+^ γδ T • Effector γδ T: Responders > Non-responders at week 4/8
n=11 HBeAg^+^ CHB Cohort (145)	PEG-IFN-α (48 weeks)	Responder (5/11): ALT normalization+ HBeAg loss+ HBV DNA reduction>3log_10_	• ↓ γδ T/Vδ2 T • γδ T_em_: Responders Non-responders
n=51 HBeAg^+^ CHB Cohort (146)	LDT (52- 112 weeks)	Responder (20/51): HBeAg seroconversion	• Peripheral CD4^-^CD8^-^ γδ T: Responders Non-responders at baseline (predicts recurrence) • ↑ Hepatic CD4^-^CD8^-^ γδ T in non-responders at week104

ALT, alanine aminotransferase; CHB, chronic hepatitis B; GrzB, granzyme B; TDF, tenofovir; LDT, telbivudine; PEG-IFN, pegylated interferon; RCT, random controlled trial.

↑, increase; ↓, decrease.

### 3.3 NKT cells

Natural killer T (NKT) cells constitute a specialized lymphocyte population distinguished by their recognition of lipid antigens presented through the CD1d molecule (103). These CD1d-restricted cells are broadly classified into two subsets, invariant NKT (iNKT) cells and diverse (type II) NKT cells. iNKT cells are characterized by a semi-invariant TCR architecture, featuring a conserved α chain rearrangement Vα24-Jα18 paired with limited β chain diversity Vβ11 in humans (103). This unique TCR configuration enables iNKT cells to detect both endogenous and exogenous lipid antigens, including the prototypical α-galactosylceramide (α-GalCer), presented via the MHC-I-like CD1d molecule (103). Additionally, iNKT cells can be activated in a TCR-independent manner through innate cytokines like IL-12 and IL-18 (147). In contrast to their invariant counterparts, type II NKT cells possess highly diverse αβ TCR repertoires while maintaining CD1d-restricted lipid antigen specificity (148). Current understanding of type II NKT cell functionality remains limited, though emerging evidence suggests their involvement in both immunoregulatory and pathogenic responses through distinct lipid antigen recognition pathways (149).

Chronic HBV infection markedly alters homeostasis and function of iNKT cells. Both peripheral and hepatic iNKT cells are significantly reduced in CHB patients compared to HCs, with negative correlation between circulating iNKT cell counts and liver injury severity (143, 150, 151). Furthermore, CD4^-^ iNKT cells are reduced in CHB, especially in those with detectable HBV DNA levels (151). Functional analyses reveal complex dysregulation of CD1d-iNKT axis in chronic HBV infection. Despite hepatic CD1d upregulation, the CD1d-iNKT system remains unactivated in CHB, showing impaired α-Galcer responses (150, 152). Surface marker profiling unveils a complex phenotype characterized by increased expression of NKG2A (153) and activation markers (CD69, CD38, HLA-DR) (150) alongside elevated exhaustion markers (Tim-3, PD-1) and reduced CD28 co-stimulation in both peripheral and hepatic iNKT cells from CHB patients compared to HCs (154). However, one study reports no significant upregulation in circulating or hepatic iNKT populations (150). Functional restoration is achieved *in vitro* through Tim-3/PD-1 blockade or CD28 activation (154). Other studies reveal that enhanced chemokine receptor expression (CCR5 and CCR6) and elevated Fas and FasL levels on peripheral iNKT cells from CHB (150). Moreover, IFN-γ^+^ NKT cells positively correlated with ALT levels and inversely correlated with HBV DNA (79, 155). Besides, other studies report diminished IL-4 and IFN-γ production in iNKT cells from CHB patients, partially reversible by exogenous IL-2 and IL-15 (150, 153, 154), while other studies find comparable cytokine production post-stimulation across disease phases upon stimulation of α-GalCer and PMA (156, 157).

Antiviral therapies elicit heterogeneous modulation of NKT cells (**Table 4**). PEG-IFN-α add-on TDF therapy reduces peripheral iNKT frequencies without altering cytokine profiles (IFN-γ and TNF-α) (143). However, other studies observe that PEG-IFN-α or LDT monotherapy conversely increase iNKT frequencies (156, 158). Notably, baseline iNKT frequencies predict sustained response to PEG-IFN-α monotherapy in HBeAg-positive patients (158). ETV treatment differentially modulates iNKT subsets, enhancing IFN-γ^+^ while reducing IL-4^+^ iNKT cells during six-month treatment (155). LDT therapy selectively reduces peripheral CD3^+^CD56^+^ NKT-like cells in treatment responders instead of non-responders (97). Longitudinal analyses further reveal post-treatment expansion of circulating CD4^−^ iNKT subsets, with baseline elevations in the CD4^−^/CD4^+^ iNKT cell ratio correlating with HBeAg seroconversion (156). Novel immunotherapies reveal distinct mechanisms. Oral HBV envelope proteins trigger a >2-fold increase in iNKT frequency alongside improved histology and seroconversion (159), while α-GalCer administration transiently suppresses total NKT cells at 2 days post-injection (recovering by day 7) and drives a shift toward CD8^+^ predominance, most prominently at the 1 μg/kg dose (101). Collectively, these findings highlight the heterogeneity of NKT cell responses across therapeutic regimens, emphasizing NKT cells as potential biomarkers for therapeutic stratification and outcome prediction in CHB management.

**Table 4:** Effects of anti-HBV therapies on NKT cells.

Population study type	Intervention	Clinical outcome	Key immunological findings
n=30 CHB (on TDF, virally suppressed): 10 add-on Peg-IFN-α; 20 on-TDF RCT (143)	TDF ± PEG-IFN-α (48 weeks)	Add-on group: HBsAg reduction	• Add-on group: ↓iNKT cell count (week 12) • Both groups: IFN-γ,TNF-α production of iNKT→
n=63 HBeAg+ CHB (ALT 2-10× ULN) Cohort (158)	PEG-IFN-α (48 weeks)	• Significant effect (26/63): HBV DNA negative+ HBeAg loss+ ALT normal • Effect (11/63): HBV DNA reduction>2log_10_; • No effect (16/63): HBV DNA reduction <2log_10_ +no HBeAg loss	• Significant effect group vs. effect/no-effect group: -↑ Peripheral NKT cells (baseline- treatment- follow-up)
n=21 HBeAg+ CHB Cohort (155)	ETV (6 months)	HBV DNA, ALT reduction	• ↑ IFN-γ^+^ iNKT • ↓ IL-4^+^ iNKT
n=41 HBeAg+ CHB (ALT 2-5×ULN) Cohort (97)	LDT (36 weeks)	HBV DNA, ALT/AST reduction Well responder (14/36): HBV DNA negative+ HBeAg seroconversion	• Peripheral NKT-like(CD3^+^ CD56^+^) - ↓ in well-responders - → in non/partial responders
n=19 HBeAg+ CHB (ALT >ULN) Cohort (156)	LDT (52 weeks)	HBeAg seroconversion (7/19)	• ↑ Circulating iNKT cells (CD4^-^ subset dominant) • Baseline CD4^-^/CD4^+^ iNKT ≥1 → higher HBeAg seroconversion
n=42 CHB cohort (159)	p.o. with HBV envelope proteins (HBsAg+preS1+preS2), every other day (20-30 weeks)	HBV DNA reduction in 35.7% of patients HBsAg/HBcAg biopsy scores improved in 41%/57.1% of patients Histological improvement (liver necroinflammatory score) in 12/40 5/19 HBeAg seroconversion	• ↑ Peripheral iNKT cells (> 2-fold)
n=27 CHB Phase I/II RCT (101)	α-GalCer at doses of 0.1/1/10 ug/kg All received 3 doses (week0, 4, 8)	No clearly affect HBV DNA and ALT levels	• ↓NKT cells at 2 days post-injection, recovery at day 7 •↓ CD4^+^ NKT cells decreased and ↑CD8^+^ NKT counterpart, most significant in 1ug/kg dose

ALT, alanine aminotransferase; AST, aspartate aminotransferase; CHB, chronic hepatitis B; ETV, entecavir; LDT, telbivudine; PEG-IFN, pegylated interferon; RCT, random controlled trail; TDF, tenofovir disoproxil fumarate; ULN, upper limit of normal.

↑, increase; ↓, decrease.

## 4 Conclusion

**Figure 1:**
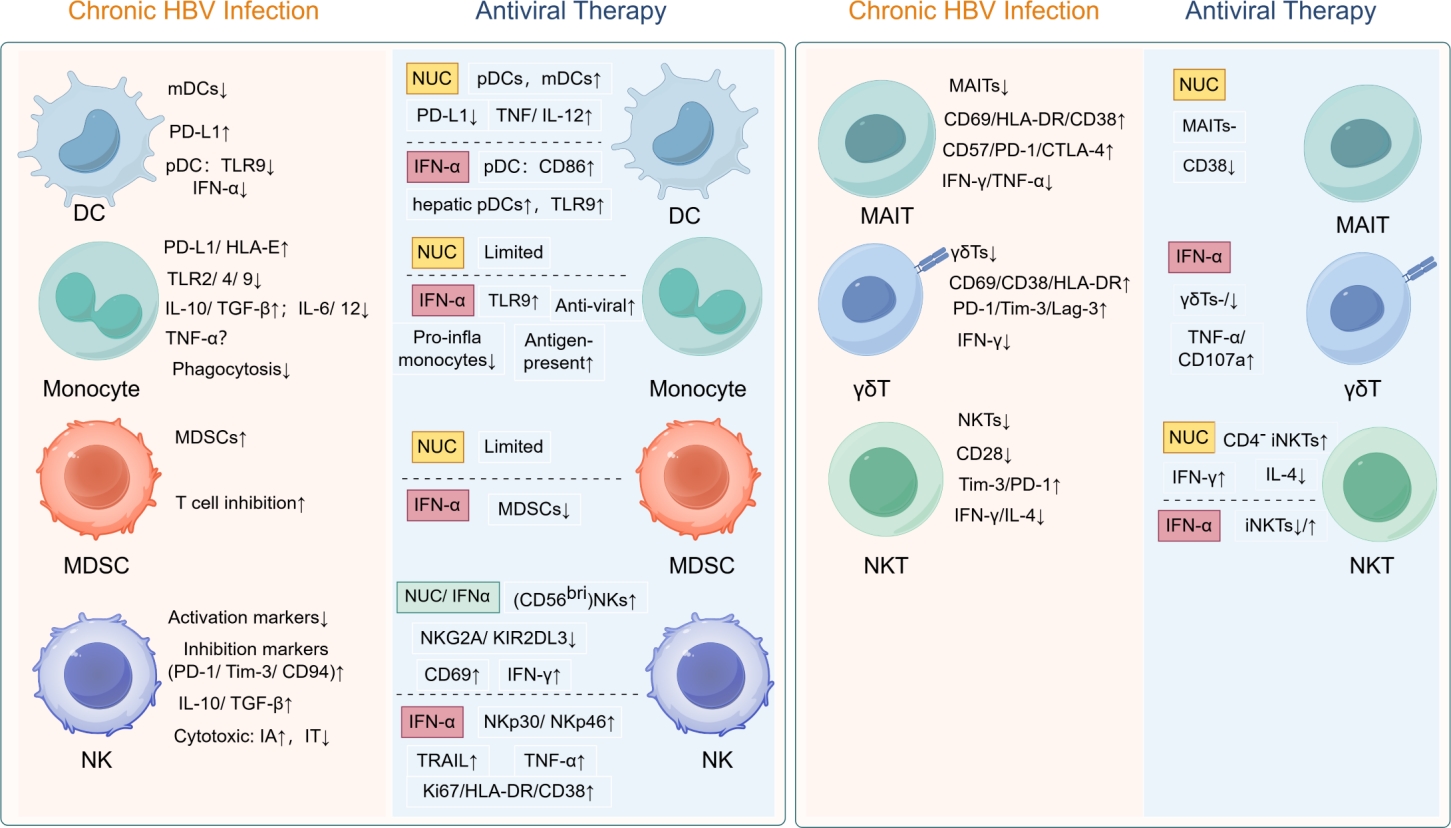
Treatment-induced immune reconstitution in chronic HBV: restoring functionality of dysregulated innate immune and unconventional T cells. HBV, hepatitis B virus; DC, dendritic cell; pDC, plasmacytoid dendritic cell; mDC, myeloid dendritic cell; PD-L1, programmed death ligand-1; TLR, Toll-like recptor; IFN, interferon; HLA, human leukocyte antigen; IL, interleukin; TGF-β, transforming growth factor-beta; TNF-α, tumor necrosis factor-alpha; MDSC, myeloid-derived suppressor cell; NK, natural killer cell; PD-1; programmed cell death protein-1; Tim-3, T-cell immunoglobulin and mucin-domain containing-3; IA, immune active phase; IT, immune tolerant phase; NKG2A, natural killer group 2 member A; KIR2DL3, killer cell immunoglobulin like receptor, two Ig domains and long cytoplasmic tail 3; TRAIL, TNF-related apoptosis-inducing ligand; CTLA-4, cytotoxic T-lymphocyte-associated protein 4; Lag-3, lymphocyte-activation gene 3; MAIT, mucosal-associated invariant T cell; NKT, natural killer T cell.

Chronic HBV infection induces broad immune dysfunction across innate (DCs, monocytes, MDSCs, NK cells) and unconventional T cell populations (MAIT, γδ T, NKT cells), characterized by inhibitory receptor upregulation, suppressed cytotoxicity, and immunosuppressive cytokine profiles (**Figure 1**). While NUCs demonstrate limited immunorestorative capacity, PEG-IFN-α exhibits superior efficacy in reversing DC/monocyte dysfunction, reducing MDSC accumulation, and partially restoring NK/unconventional T cell activity (**Figure 1**). Critically, the currently limited evidence base (summarized in **Tables 1**-**4**) reveals a paucity of prospective studies tracking innate immune dynamics during NUC therapy, hindering comprehensive understanding of functional restoration in these compartments. Future studies should prioritize intrahepatic immune profiling, given the profound functional and phenotypic disparities between circulating and liver-resident immune cells in chronic HBV infection.

Emerging immunomodulatory agents show promise in restoring antiviral immunity. For instance, TLR agonists like selgantolimod (TLR8 agonist) remodel the intrahepatic immune microenvironment by activating MAIT and NK cells (160). Combination therapies pairing immunomodulators (anti PD-1/PD-L1, TLR agonists, therapeutic vaccines and monoclonal antibodies) and viral-targeting agents (siRNA, core protein allosteric modulators (CpAMs) and virus entry inhibitors) represent a theoretically powerful strategy to overcome monotherapy limitations in achieving HBV functional cure (161). While several clinical studies confirm the efficacy of such combinations (162–164), their underlying immune mechanisms remain inadequately explored. The success of combination strategies will likely depend on identifying immunological biomarkers and implementing high-dimensional immune profiling to enable precise patient selection (165, 166). In summary, advancing immune-focused combinatorial regimens within precision medicine frameworks is essential to overcome HBV’s potent immunosuppressive mechanisms.

## Glossary

ADCC: Antibody-Dependent Cellular Cytotoxicity
ADF: Adefovir Dipivoxil
AHB: Acute Hepatitis B
AKT: Protein Kinase B
ALT: Alanine Aminotransferase
Arg1: Arginase-1
AST: Aspartate Aminotransferase
AVT: Antiviral Therapy
BDCA-2: Blood Dendritic Cell Antigen-2
BTN3A1: Butyrophilin Subfamily 3 Member A1
CCL25: C-C Motif Chemokine Ligand 25
CCR5/CCR6: C-C Chemokine Receptor Type 5/6
CD: Cluster of Differentiation
cDC: Conventional Dendritic Cell
CHB: Chronic Hepatitis B
CTLA-4: Cytotoxic T-Lymphocyte-Associated Protein 4
CX3CR1: CX3C Chemokine Receptor 1
DAAs: Direct-Acting Antiviral Agents
DBIL: Direct Bilirubin
DC: Dendritic Cell
ERK: Extracellular Signal-Regulated Kinase
ETV: Entecavir
FDC: Follicular Dendritic Cell
gMDSC: Granulocytic Myeloid-Derived Suppressor Cell
GZ: Gray Zone
HBeAg: Hepatitis B e Antigen
HBsAg: Hepatitis B Surface Antigen
HBV: Hepatitis B Virus
HCC: Hepatocellular Carcinoma
HC: Healthy Controls
HLA: Human Leukocyte Antigen
IA: Immune-Active Phase
IC: Inactive Carrier Phase
IFN: Interferon
IFNAR2: Interferon Alpha/Beta Receptor Subunit 2
IL: Interleukin
IT: Immune-Tolerant Phase
KIR: Killer-Cell Immunoglobulin-Like Receptor
LAG-3: Lymphocyte-Activation Gene 3
LAM: Lamivudine
LDT: Telbivudine
LY6E: Lymphocyte Antigen 6E
MAIT: Mucosal-Associated Invariant T Cells
mDC: Myeloid Dendritic Cell
MDSC: Myeloid-Derived Suppressor Cell
MFI: Mean Fluorescence Intensity
M-MDSC: Monocytic Myeloid-Derived Suppressor Cell
moDC: Monocyte Dendritic Cell
MR1: MHC Class I-Related Gene Protein
MyD88: Myeloid Differentiation Primary Response 88
NF-κB: Nuclear Factor Kappa-Light-Chain-Enhancer of Activated B Cells
NK: Natural Killer Cells
NKG2A/D: Natural Killer Group 2 Member A/D
NKT: Natural Killer T Cells
NUC: Nucleos(t)ide Analog
OXPHOS: Oxidative Phosphorylation
PBMC: Peripheral Blood Mononuclear Cell
PD-1: Programmed Cell Death Protein 1
PD-L1: Programmed Death-Ligand 1
PEG-IFN-α: Pegylated Interferon-Alpha
pDC: Plasmacytoid Dendritic Cell
PMN-MDSC: Polymorphonuclear Myeloid-Derived Suppressor Cell
sCD163: Soluble CD163
STAT3: Signal Transducer and Activator of Transcription 3
STK4: Serine/Threonine Kinase 4
Tbet: T-box Transcription Factor TBX21
TCR: T-Cell Receptor
TDF: Tenofovir Disoproxil Fumarate
TGF-β: Transforming Growth Factor Beta
Th1: T Helper 1 Cells
Tim-3: T-cell Immunoglobulin and Mucin-Domain Containing-3
TLR: Toll-Like Receptor
TNF-α: Tumor Necrosis Factor-Alpha
TRAIL: TNF-Related Apoptosis-Inducing Ligand
ULN: Upper Limit of Normal
UTCs: Unconventional T Cells
Vδ1/Vδ2: T-Cell Receptor Delta Variable Segments.
